# Nickel-based electrochemical sensor with a wide detection range for measuring hydroxyl ions and pH sensing

**DOI:** 10.1016/j.jelechem.2021.115547

**Published:** 2021-08-15

**Authors:** Behnaz Jafari, Madhivanan Muthuvel, Gerardine G. Botte

**Affiliations:** a Chemical and Electrochemical Technology and Innovation Laboratory, Department of Chemical Engineering, Texas Tech University, Lubbock, TX 79401, USA; b Center for Electrochemical Engineering Research, Department of Chemical and Biomolecular Engineering, Ohio University, Athens, OH 45701, USA

**Keywords:** Hydroxyl ions detection, pH sensor, Nickel oxyhydroxide, Water quality, Electrochemical sensors

## Abstract

The importance of hydroxyl ions concentration measurement in solution necessitates the development of suitable and simple sensing methods for online detection. To address this need, an electrochemical sensor was developed using nickel as the working electrode that enabled specificity towards hydroxyl ions and their successfully quantification. The electrochemical reaction between nickel and hydroxyl ions in solution generates a current which is proportional to the concentration of hydroxyl ions and consequently is also correlated to the pH of the solution. Cyclic voltammetry and chronoamperometry techniques were used to prepare and activate the nickel electrode for detection. The analysis of chronoamperometric experiments in different solutions exhibited three calibration curves between the current and hydroxyl ion concentrations for three regions in the range of 0.3 µM to 4.8 M with a short response time of 14 s. Up to now, no other hydroxyl ion sensor has been reported that can reach this detection limit. This sensor also holds promise to work perfectly in highly alkaline environments where only costly commercial electrodes are available to quantify pH. The results show three linear calibration curves between the current and pH of the solution over the pH range of 7.4 to 14.6. In addition to the wide detection range, the sensor exhibits a relative standard deviation percentage lower than 7% over 9 months of experiments, confirming repeatability of measurements and stability of the electrode. The potential of the proposed method to measure pH in real applications was demonstrated by testing the sensor in solutions containing urea, nitrates, phosphates, and sulfates.

## Highlights

• Nickel-based sensor allows direct quantification of hydroxyl ions in solution.
• The nickel-based sensor is applicable in highly alkaline solutions.
• The detection range of the sensor is from 0.3 µM to 4.8 M.
• The OH ion detection limit is %94 lower than the lowest reported in the literature.
• The sensor has a rapid response time of 14 s.

## Graphical abstract

**Figure:**
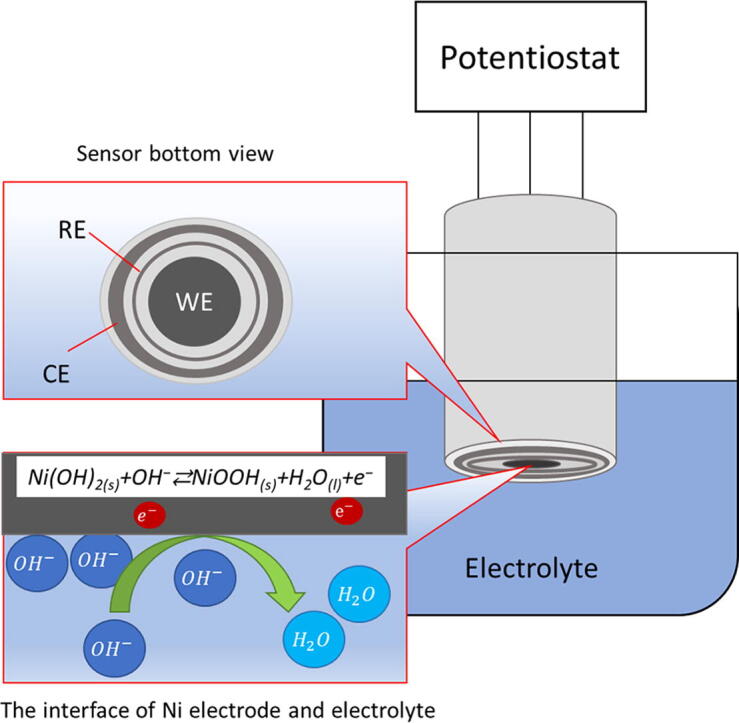

## 1 Introduction

The detection and measurement of hydroxyl ions (${\mathrm{OH}}^{-}$) in solution are crucial due to their role in many applications, such as treatments of natural water and wastewater, aquatic life, and food industries [1]. Up to now, no ideal method has been developed to directly measure ${\mathrm{OH}}^{-}$ concentrations, and most quantitative methods have focused on proton ($H^{+})$ sensing and pH measurement [2].

The quantification of $H^{+}$concentration is normally carried out by pH sensors. pH sensors, which are made of a glass electrode, are known as the premiere device for pH measurement in aqueous solutions [3]. However, they show alkaline errors at high pH values, which limit their use for the detection of hydroxyl ions [4], [5], [6]. Furthermore, they suffer from other disadvantages, namely the need for an internal solution, sluggish response, high Ohmic resistance, fragility of the glass, and miniaturization issues [7]. In recent years, electrochemical, polymer-based, and biological pH sensors have been investigated, which need complicated bulky instruments and suffer from a limited pH detection range and temperature dependence [8].

Other types of pH sensors have been reported, such as ion-selective membranes, fluorescent sensors, and metal oxide sensors. However, these sensors require constant optimization and calibration because of their operational instability [9].

With the widely increasing interest in ${\mathrm{OH}}^{-}$ measurement, several attempts have been made to develop an ${\mathrm{OH}}^{-}$ sensor. For example, a nickel oxide screen printed sensor was developed for the first time for the electroanalytical sensing of hydroxide ions. However, this sensor is only useful over a low detection range of micro-molar to millimolar [10]. Other researchers demonstrated the capacity of a reaction-based luminescent switch-on sensor using an iridium (III) complex to detect ${\mathrm{OH}}^{-}$ in simulated wastewater [11]. A third example includes an electrical sensor developed based on the impedance behavior of ${\mathrm{OH}}^{-}$ adsorbed onto amorphous InGaZnO4 (a typical inorganic metal oxide) thin film surfaces. Findings reported that the impedance of the InGaZnO4 thin film increased proportionally to the ${\mathrm{OH}}^{-}$ concentration in the solution [2]. The last two methods, the luminescent and electrical sensor, require a long fabrication process and measuring procedure. Most of ${\mathrm{OH}}^{-}$ sensing techniques reported in the literature are time consuming and need sophisticated instruments. Consequently, they cannot be employed for on-line measurements. Most of these sensors suffer short lifetimes and narrow detection ranges. Therefore, a sensor directly targeting ${\mathrm{OH}}^{-}$ over a wide detection range, with a short response and long lifetime seems essential for advanced ${\mathrm{OH}}^{-}$ measurement.

In this paper we developed and tested an electrochemical nickel-based sensor that directly detects ${\mathrm{OH}}^{-}$. This sensor generates a current signal correlated to the ${\mathrm{OH}}^{-}$ concentration as well as the pH of the solution. The sensor signal is based on the current produced by the following reaction between the nickel (Ni) electrode and the hydroxyl ions present in the solution:

(1) Ni(OH)_2(s)_ + OH^−^ ⇄ NiOOH_(s)_ + H_2_O_(l)_ + e^−^

Eq. (1) shows the oxidation reaction of nickel hydroxide (*Ni(OH)_2_*) to nickel oxyhydroxide (*NiOOH)* and the change in the valence of metallic nickel in alkaline media from +2 to +3 [12]. As this reaction suggests, the generated current is proportional to the hydroxyl ion concentration in the solution, so nickel electrodes could selectively determine the ${\mathrm{OH}}^{-}$ concentration. It is important to define the applied cell potential range of the sensor as the same electrode configuration is also known for enabling the oxidation of organic compounds found in wastewater, such as urea [13], [14], [15].

The analysis of the nickel reactions was evaluated by Cyclic Voltammetry (CV). Chronoamperometry was used to find the correlation between the current and ${\mathrm{OH}}^{-}$ concentration, and the current and solution pH. Moreover, the sensor was tested in solutions containing urea, nitrates, phosphates, and sulphates to evaluate the interference of these ions with the sensor response and investigate the potential application of the sensor for water and wastewater monitoring.

## 2 Material and methods

### 2.1 Chemicals and apparatus

Potassium hydroxide (KOH) pellets (≥85%, CAS 1310-58-3), sodium hydroxide (NaOH) pellets (98.7%, CAS 1310-73-2), urea (99.8%, CAS 5713-6), sodium dihydrogen phosphate (99%, CAS 7558-80-7), sodium nitrate (≥95%, CAS 7631-99-4), and ammonium sulfate (99.95%, CAS 7783-20-2) were supplied by Fisher Scientific. Ultrapure water (>18 MΩ) was used throughout testing. Thermo Scientific Orion pH buffers (pH 4.01, pH 7.00, and pH 10.01) and Cole-Parmer Oakton buffer solution (pH 12.46) were purchased from Fisher Scientific.

**Fig. 1:**
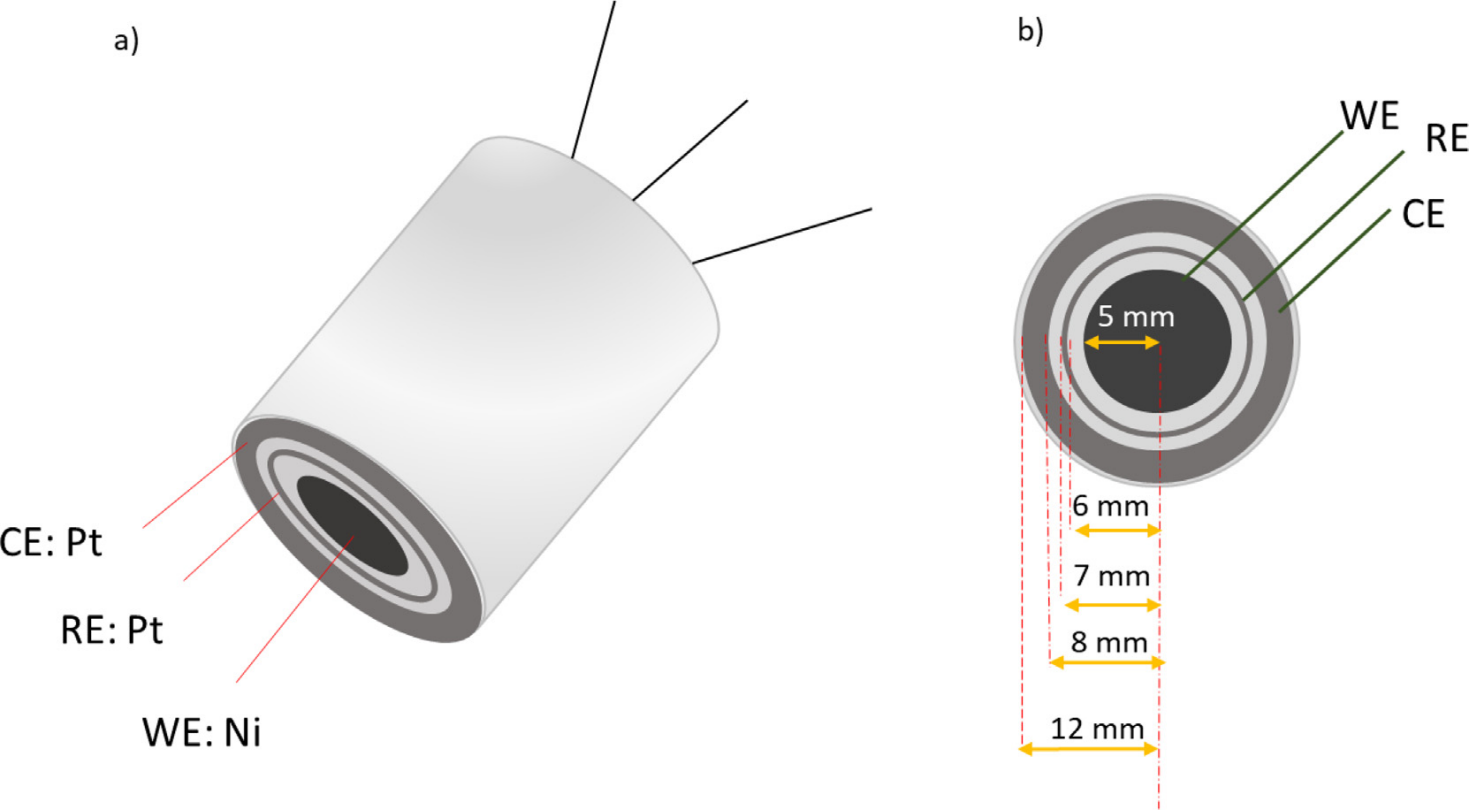
Schematic of **a)** the nickel-based sensor, and **b) the** bottom view of the sensor. A three-electrode system consisting of the working electrode, reference electrode, and counter electrode was used as the ${OH}^{-}$ sensor.

The sensor was designed as a concentric three-electrode system (Fig. 1) consisting of Ni foil (McMaster-Carr, 200 nickel, over 98% pure, CAS 7440–02-0, 0.127 mm thickness) as the working electrode (WE) in the center and two platinum (Pt) rings (Espi Metals, 99.95%, CAS 7440–06-4, 0.127 mm thickness) as the pseudo-reference electrode (RE) and counter electrode (CE). All three electrodes were embedded into an acrylic case (McMaster-Carr, Clear-scratch, and UV resistant acrylic rod, 25.4 mm diameter, 33 mm height) and only their one circular surface was exposed to the electrolyte. Ni wires (Alfa Aesar, 99.5%, CAS 7440–02-0, 1 mm diameter) were spot welded on the electrodes as current collectors using a Miller® resistance spot welder (SSW-2020ATT) operating at 9 A per second.

A Solartron 1470 E Potentiostat was employed for all electrochemical measurements. Titration tests were performed using a Mettler Toledo G20 compact titrator according to Standard Methods [16] to determine the ${\mathrm{OH}}^{-}$ level of the solution. An Acorn pH 5 m from Oaktan instruments was used to measure pH values of each solution.

### 2.2 Procedure

**Fig. 2:**
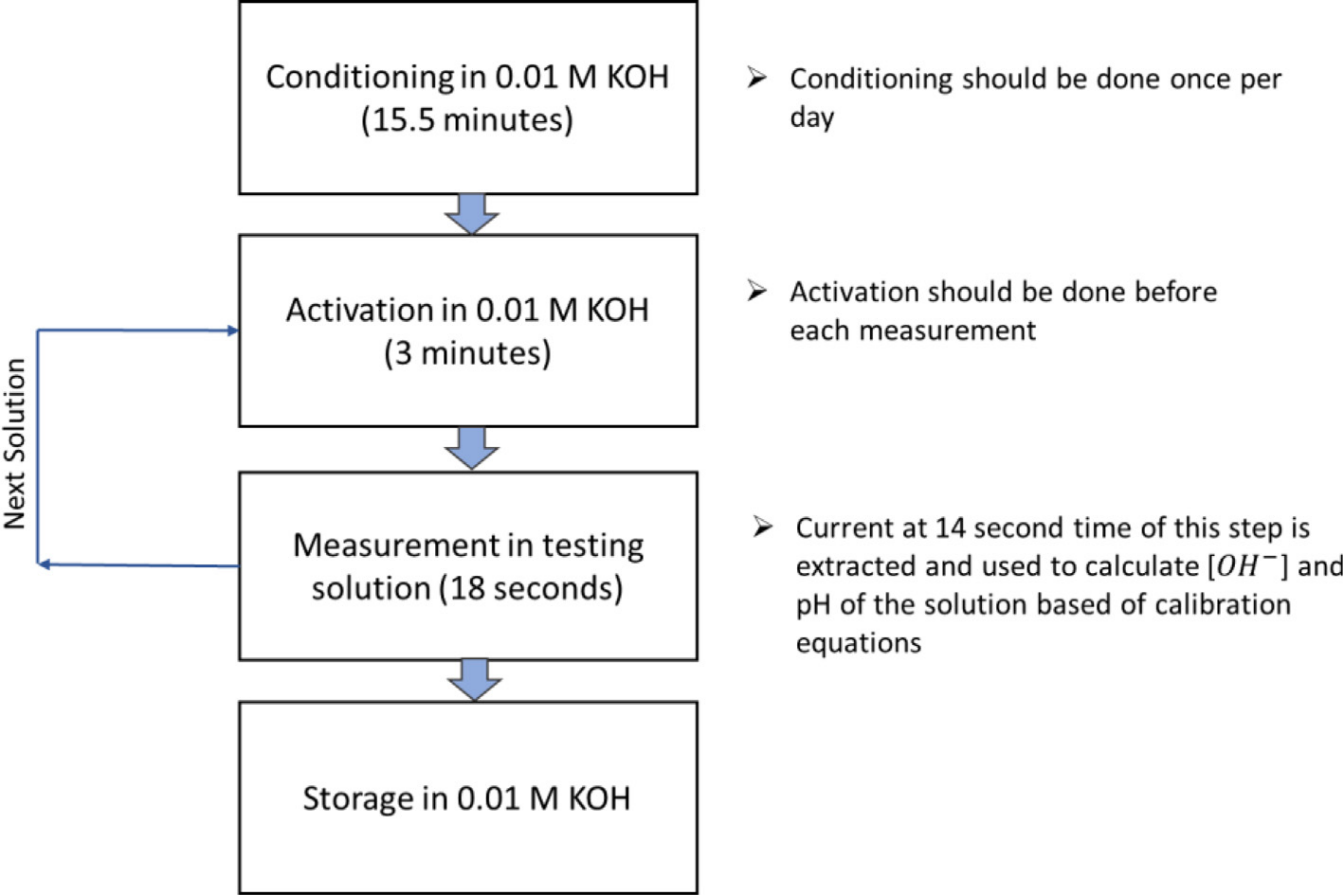
Schematic representation of the standard operating procedure for the nickel-based sensor. Steps include conditioning, activation, measurement, and storage for preservation of the sensor.

The procedure to perform measurements using the nickel-based sensor is depicted in Fig. 2. It consists of conditioning, activation, and measurement steps. As this figure shows, the sensor is stored in a solution of 0.01 M KOH after doing measurements to preserve its activity. Details about each step are described in the next sections.

#### 2.2.1 Conditioning

**Fig. 3:**
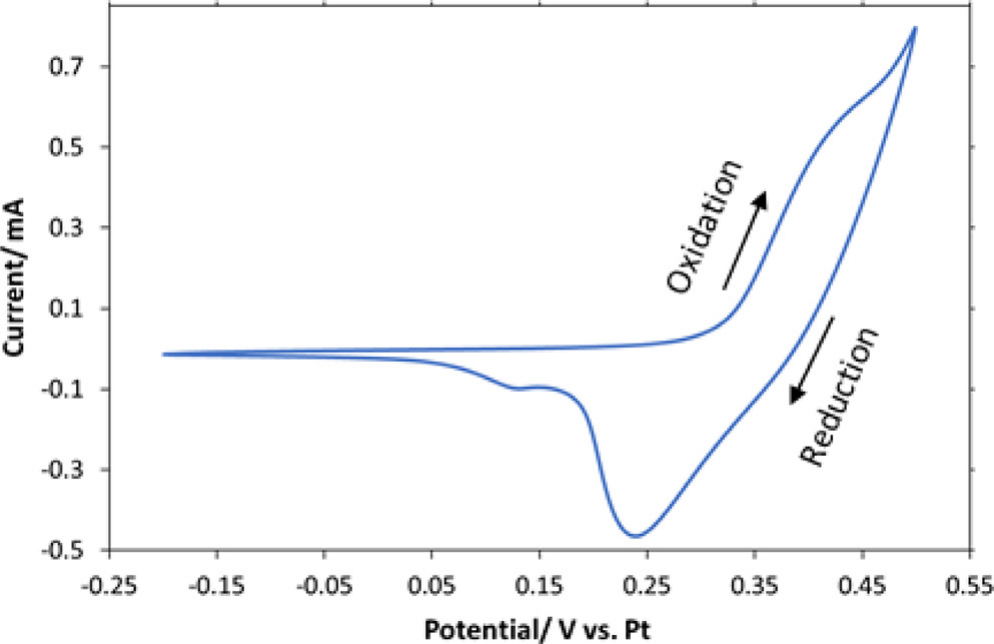
Cyclic voltammogram spectra of Ni in 0.01 M KOH solution at room temperature. Cyclic voltammetry was employed for conditioning of the working electrode surface in a potential range from −0.2 V to 0.5 V vs. Pt reference at a scan rate of 15 mV$s^{-1}$.

The first step was to form a stable nickel surface for the detection of ${\mathrm{OH}}^{-}$. Cyclic Voltammetry was performed in 0.01 M KOH in a potential range from −0.2 V to 0.5 V vs. the reference electrode at a scan rate of 15 mV$s^{-1}$ for 10 cycles (15.5 min) to reach a stable Ni surface and facilitate catalyst formation. Fig. 3 shows a typical cyclic voltammetry curve of Ni in 0.01 M KOH versus the Pt reference electrode.

It was observed that after 5 cycles, the CVs begin to overlap. This indicated that the Ni electrode reached a stable surface and was ready for catalyst formation, which in our process is called activation step. In Fig. 3, the anodic peak for Ni^2+^ oxidation to Ni^3+^ was detected at 0.44 V vs. Pt at a scan rate of 15 mV s^−1^. In the reverse scan, two reduction peaks were observed corresponding to the Ni^3+^ reduction to Ni^2+^. During the reduction sweep, the major cathodic peak at 0.24 V vs. Pt represents the reduction of β-Ni(OH)_2_/β-NiOOH redox couple and the second peak has been assigned to the reduction of α-Ni(OH)_2_/γ-NiOOH at 0.13 V vs. Pt [12], [17], [18], [19]. In order to maintain the same redox properties of the Ni electrode for all tests, the conditioning step should be performed once per day in 0.01 M KOH. It was observed that once the conditioning step was done, at least 30 tests could be performed in one day without the need to repeat that step.

#### 2.2.2 Activation

Based on Eq. (1), it is hypothesized that Ni^2+^ works as the catalyst to detect ${\mathrm{OH}}^{-}$. Therefore, the next step after the CV (conditioning) was to activate the nickel working electrode and generate Ni^2+^ as the catalyst for ${\mathrm{OH}}^{-}$ measurement.

**Fig. 4:**
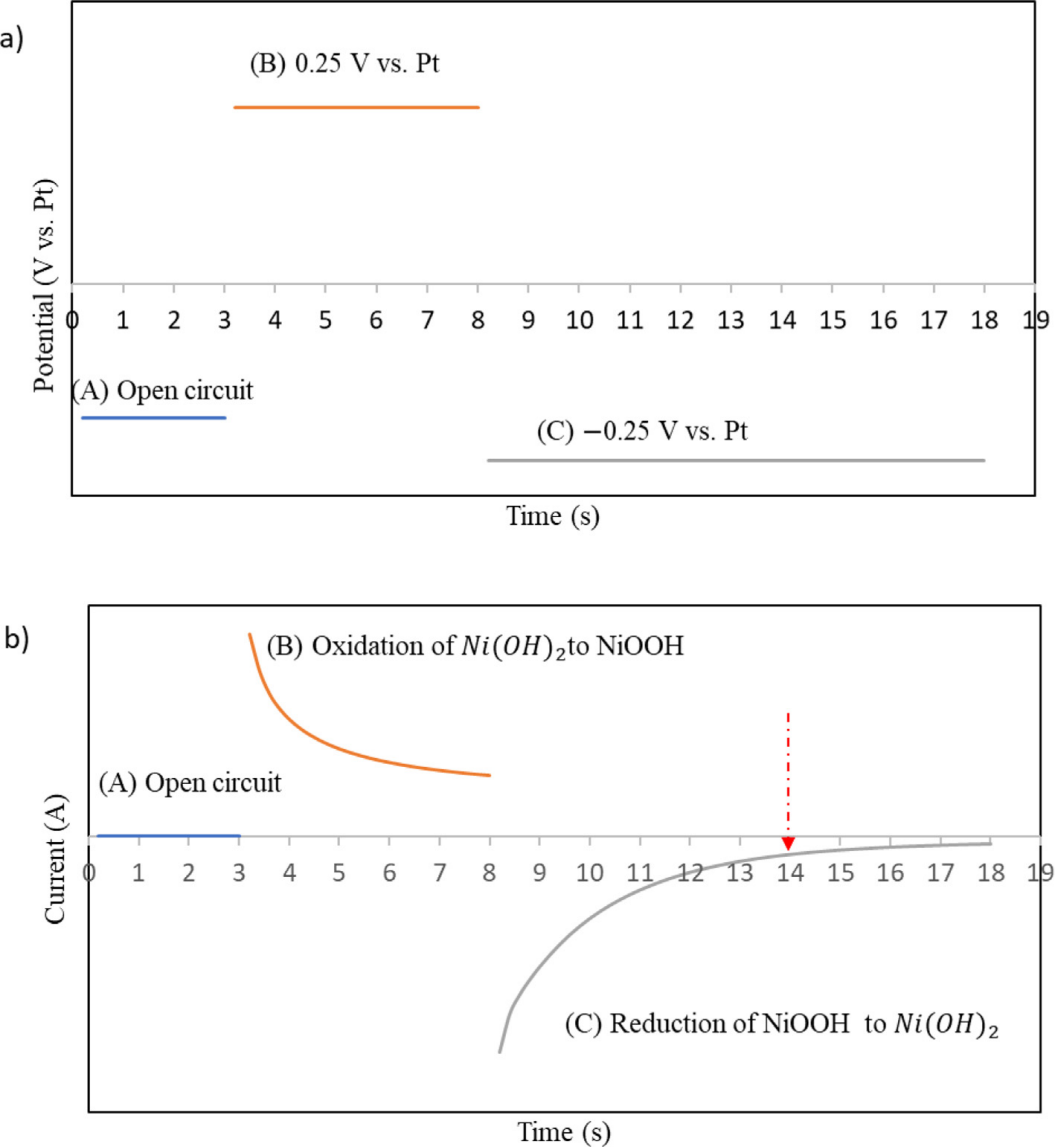
**a)** Schematic of input potential pulse for chronoamperometry tests A) open circuit for 3 s, B) 0.25 V vs. Pt for 5 s, and C) −0.25 V vs. Pt for 10 s. Steps A, B, and C are considered one cycle. For the activation step, 10 cycles were performed in 0.01 M KOH to create a stable layer of Ni^2+^ at the surface of the working electrode. For the measurement step, one cycle of the potential pulse was run to find the output current. **b)** Schematic of the output current response of the measurement step. As it is marked on Fig. 4b, the current at 14 s was used to develop the calibration curves.

Chronoamperometry was performed in 20 ml of 0.01 M KOH to synthesize a stable catalyst layer on the electrode. To run chronoamperometry tests, a potential pulse was applied to the sensor as follows: **A)** the pulse started with an open circuit for 3 s; at the open circuit potential, nickel converted to nickel hydroxide [12]. **B)** the voltage was stepped to 0.25 V vs. Pt and was held at this voltage for 5 s to oxidize Ni(OH)_2_ to NiOOH. **C)** the voltage was stepped down to −0.25 V vs. Pt and was held for 10 s to reduce NiOOH to Ni(OH)_2_. The schematic of this potential pulse is shown in Fig. 4a. If the steps from A to C are considered as one cycle, then 10 cycles are required to form the active catalyst, which took 3 min in total. Through pulsing the potential between these three steps, a stable layer of Ni^2+^ at the surface of the working electrode was created.

#### 2.2.3 Measurement

To perform the measurement, the sensor was immersed in 20 ml of each testing solution of different KOH concentrations at room temperature. Chronoamperometry tests were performed to measure current for each solution by applying the same potential pulse with the activation step (shown in Fig. 4a). It should be noted that for the measurement step only one cycle of the potential pulse was required, which took 18 s. As shown in Fig. 4b, the current responses at 14 s of the measurement step were used to form calibration curves. A good distinction between the reduction currents was observed at this specific time along with a linear correlation between the current and pH of the solution. In addition, at this specific time, the current responses seemed to be stable.

#### 2.2.4 Storage

The sensor storage solution was 0.01 M KOH, and its use ensured that the Ni electrode remained at the same redox state until the next use. In addition, storing the sensor in 0.01 M KOH facilitated the standard operating procedure, since the sensor conditioning and activation steps were performed in the 0.01 M KOH as well. Therefore, these steps can be done before each test, and the sensor will be prepared/ready for each measurement.

## 3 Results and discussion

### 3.1 Sensor chronoamperometric response in alkaline media

To create calibration curves, solutions of different ${\mathrm{OH}}^{-}$ concentrations using KOH were prepared and used as test media. The automatic titrator and the commercial pH meter were used to determine the ${\mathrm{OH}}^{-}$level and pH value of the solutions, respectively.

**Fig. 5:**
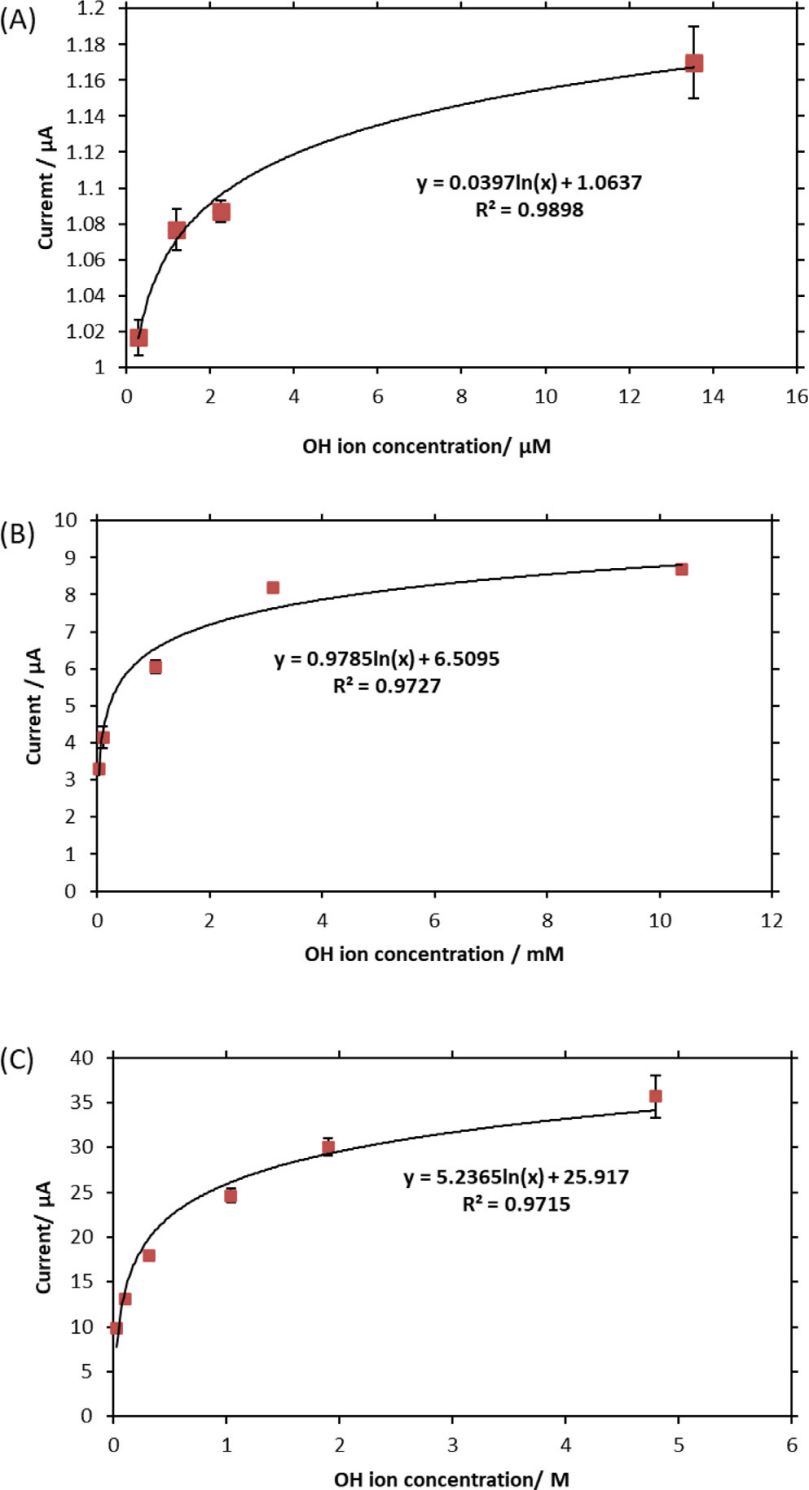
Correlation between current and ${OH}^{-}$ concentration in KOH solutions ranging from (A) 0.3 µM to 14 µM (B) 0.03 mM to 10.4 mM (C) 0.03 M to 4.8 M. The currents at 14 s of measurement step for each solution were used to develop the calibration curves. In all three equations shown on Fig. 5, y represents current, and its unit is µA and × represents OH ion concentration and its unit is µM (Fig. 5A), mM (Fig. 5B), and M (Fig. 5C).

Analysis of the chronoamperometry measurements revealed a trend in current responses. As it was mentioned earlier, the output current at 14 s time was extracted for all measurements which resulted in three calibration curves: the first from 0.3 µM to 13.5 µM, the second from 31 µM to 10.4 mM, and the third from 31 mM to 4.8 M. Fig. 5A, B, and C show the mathematical correlations between the current and ${\mathrm{OH}}^{-}$ concentration for each region. It should be noted that all data points are the average of three measurements performed in each test solution.

**Fig. 6:**
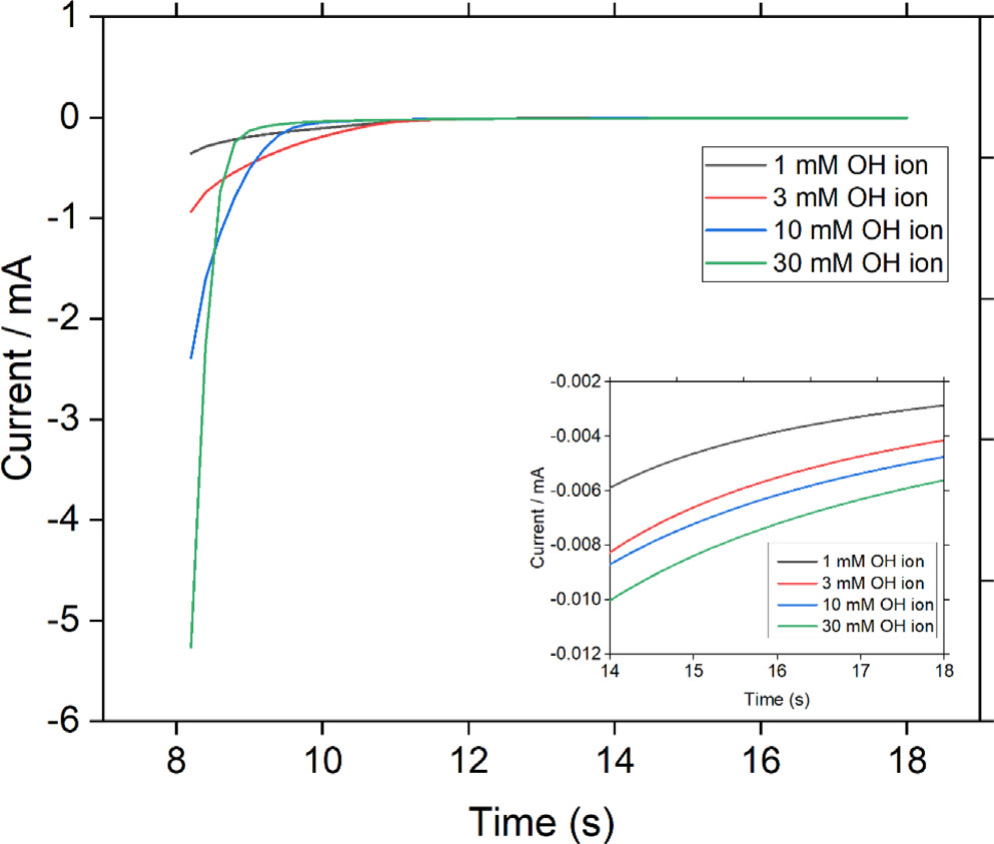
Reduction Current vs. time for solutions of different ${OH}^{-}$concentrations. Currents are stable at elapse times above 14 s.

Fig. 6 (including enlarged view) demonstrates why currents at 14 s were selected to create calibration curves. In this figure, the current vs. time is shown for four solutions with different ${\mathrm{OH}}^{-}$ concentrations. For <14 s, the current responses do not show a consistent trend. The possible explanation for the current behavior is that higher ${\mathrm{OH}}^{-}$ concentration, results in the built up of electrostatic charge in the double layer. The double layer charge decays faster for the more concentrated solution. Then, the current gets stable, where it is dominantly produced from the reduction of ${\mathrm{Ni}}^{3+}$ and diffusion of ${\mathrm{OH}}^{-}$ to the bulk. In this region (elapsed time > 14 s), the current consistently increases with increasing the ${\mathrm{OH}}^{-}$ concentration.

As results confirmed, the nickel-based electrochemical sensor offers a wide detection range (from 0.3 µM to 4.8 M) and a short response time (14 s). The minimum detectable concentration of ${\mathrm{OH}}^{-}$ with the sensor was found to be 0.3 µM. Table 1 provides a comparison between the detection limit of recent ${\mathrm{OH}}^{-}$ sensing techniques. The nickel-based sensor offers a better detection limit compared to previous methods; and its detection range covers concentrated alkaline solutions where costly commercial electrodes are required for measurement.

**Table 1:** Overview of the detection limit of different ${OH}^{-}$ sensing methods. The nickel-based sensor has the most superior limit of detection.

Sensing Method	Detection Limit	Reference
Nickel-based electrochemical sensor	0.3 µM	Present study
Electrical sensor based on amorphous InGaZnO4 thin film	100 mM	[2]
Reaction-based luminescent sensor using iridium (III) complexes	4.96 µM	[8], [11]
Nickel oxide screen printed electrodes	23 µM	[10]
Gold ultra-micro electrode arrays	10 µM	[20]

Several KOH solutions with different concentrations of ${\mathrm{OH}}^{-}$ were tested, and the current for each was collected. The current taken at 14 s was placed into the calibration equations to find the ${\mathrm{OH}}^{-}$ concentration of each solution. Table 2 shows the calculated values by the nickel-based sensor in comparison with the measurements made by titration. The relative standard deviation percentage (RSD %) was found to be lower than 7% for ${OH}^{-}$ nickel-based sensor, indicating sufficient repeatability throughout the measurements. Our experiments with the automatic titrator show an average of 1% RSD. Compared to the automatic titrator, this sensor does not improve the repeatability, but it improves the detection range and shortens and simplifies the detection procedure. We envision that through optimization of the procedure, such as creating more active catalyst or narrowing the test time, we can improve the repeatability of the sensor.

**Table 2:** Comparison of hydroxyl ion concentration in KOH solutions using titration and the nickel-based sensor.

Titration	Nickle-based sensor	
${\mathrm{OH}}^{-}$ concentration (M)	${\mathrm{OH}}^{-}$ concentration (M)	Relative standard deviation %
3.06$\times 10^{-4}$	2.18$\times 10^{-4}$	6.71
4.32$\times 10^{-3}$	7.45$\times 10^{-3}$	6.83
3.12$\times 10^{-2}$	2.81$\times 10^{-2}$	6.44
1.04$\times 10^{-1}$	0.79$\times 10^{-1}$	5.57

**Fig. 7:**
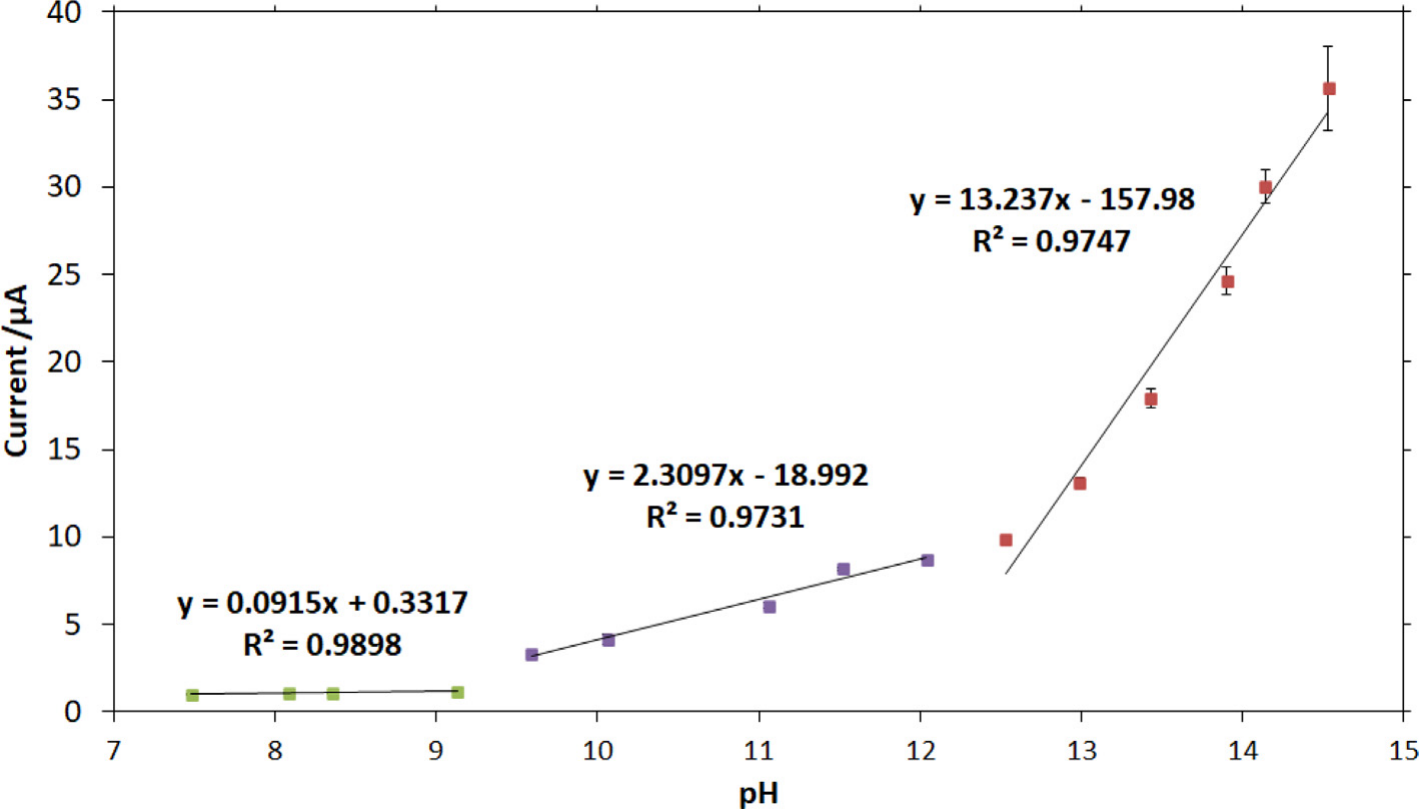
Correlation between the current and the solution pH. Three linear regions were observed in alkaline media: the first over the pH range of 7.4–9.1, the second over the pH range 9.4–12, and the third over the pH range 12.4–14.7. The broad detection range and excellent linearity confirm that this sensor could be used for pH measurements. In all three equations shown in the Fig. 7, y represents current in µA and x represents pH.

In addition to ${\mathrm{OH}}^{-}$ concentration calibration curves, the analysis of the chronoamperometric responses led us to a linear correlation between the current and the solution pH. As Fig. 7 depicts, the measurement of ${\mathrm{OH}}^{-}$ concentration can allow the pH of the solution under investigation to be determined. This suggests that the nickel-based sensor could be utilized in extreme conditions where the pH glass electrode is non-operational.

The sensitivity (S) of the sensor can be calculated according to the following equation:

$$S=\frac{d(current)}{d(pH)}$$

Based on Fig. 7, the detection sensitivity (slope factor) is **0.0915 µA/pH** over the pH range of 7.4–9.1, **2.3097 µA/pH** over the pH range of 9.4–12, and **13.237 µA/pH** over the pH range of 12.4–14.7. The sensor offers higher sensitivity over more concentrated alkali ranges. We anticipate that further activation of the nickel electrode could increase the sensitivity of the sensor.

### 3.2 Sensor performance in measuring pH of standard buffer solutions

The sensor performance was further assessed by testing pH 10.01 and pH 12.46 standard buffer solutions, which are within the pH range of detection. Table 3 shows the results of these experiments. The measurements were repeated for three consecutive days, three replicates each day to verify the repeatability of the sensor. The last column represents the RSD% over three values obtained by the nickel-based sensor over three days. The results verify the reliable performance of the sensor for measuring pH of the standard buffer solutions using the calibration curves developed for KOH solutions. Negligible variation (<2%) of the measurements demonstrate an excellent repeatability of the nickel-based sensor.

**Table 3:** Determination of pH of buffer solutions using the pH calibration curves developed for the nickel-based sensor.

Buffer Solution	Day 1	Day 2	Day 3	RSD %
pH 10.01	10.36	10.07	10.14	1.21
pH 12.46	12.24	12.52	12.12	1.36

### 3.3 Sensor application to measure pH of mixed solutions

To further study the behavior of the sensor and evaluate the interference of other ions, the sensor was applied to determine the pH in simulated untreated blackwater. Table 4 demonstrates the preliminary design parameters to mimic the target environment. Potassium hydroxide solutions mixed with urea, ammonium sulfate, sodium nitrate, and sodium dihydrogen phosphate in the pH range of 7–9 were prepared and used as the control environment. The concentration of these species, which is shown in Table 4, was kept constant and the ${\mathrm{OH}}^{-}$ concentration was varied to adjust pH in the range of 7–9. The procedure described for conditioning, activation, and measurement steps (Section 2.2) was followed, and the current responses were collected for different solutions as explained in the previous sections.

**Table 4:** Mixed solutions composition. The constituents and their composition were selected to evaluate the effect of other anions on the sensor response and the sensor performance in real applications.

Parameter	Concentration (ppm)
Urea	20,000
Ammonium Sulfate	200
Sodium Nitrate	600
Sodium dihydrogen Phosphate	50
pH	7–9

**Fig. 8:**
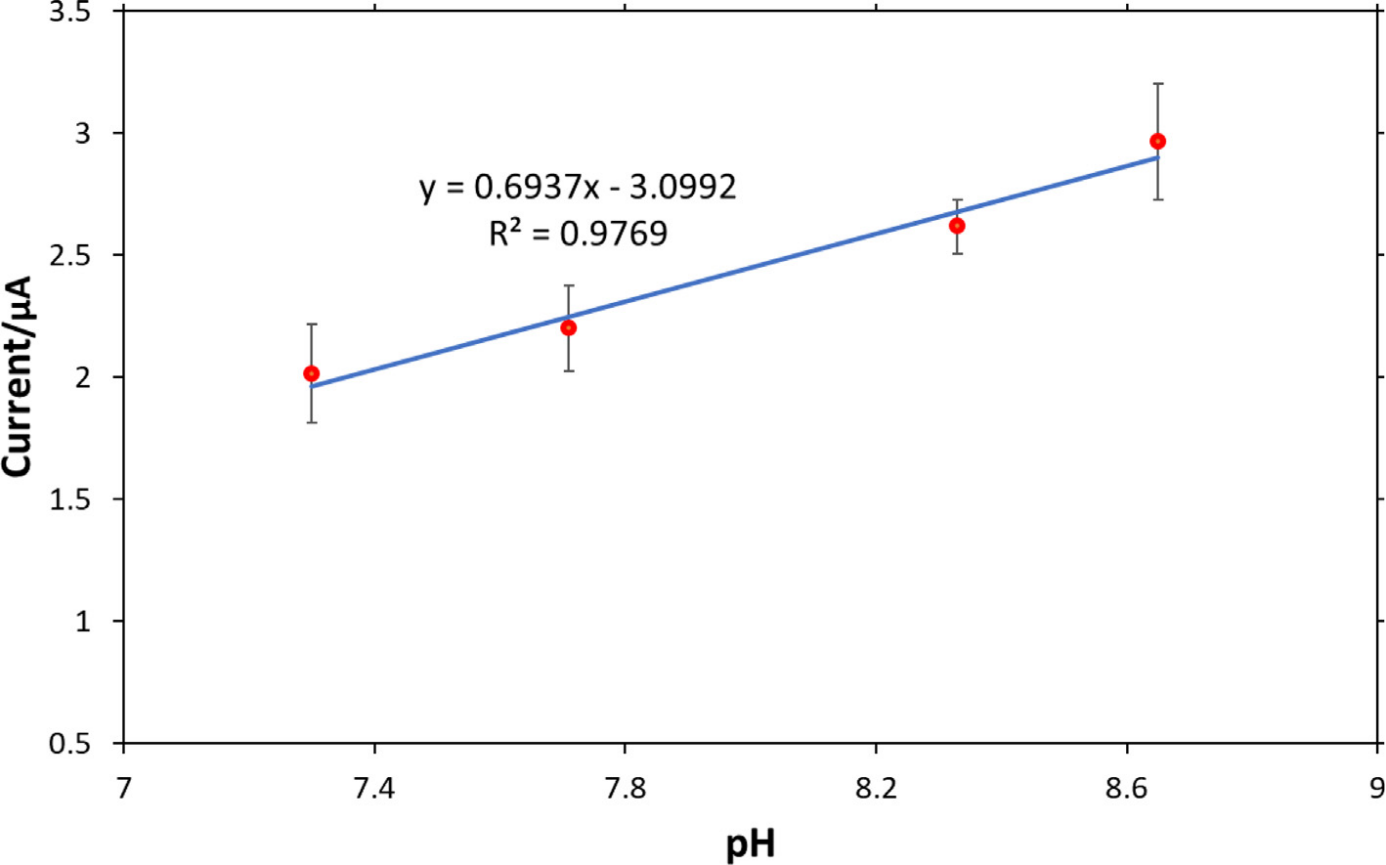
Correlation between current response of the sensor and pH in mixed solutions in the pH range of 7–9. The linear calibration curve in the mixed solution shows promising results to use this sensor in complex environments. In the equation shown on the graph, y represents current in µA and x represents pH.

The presence of urea could have a significant effect on the measurements, since urea electrolysis is catalyzed in the presence of Ni^3+^ in alkaline media [14] and could interfere with the sensor response. However, as Fig. 8 shows, despite the presence of high concentrations of urea and other anions in the solution, the current response of the sensor in mixed solution was found to be proportional to the pH of the solution and obeyed a linear relationship. A possible explanation for why we did not observe significant alteration in the measurements and the calibration curve is that the pulse applied in the measurement step did not provide the potential required to oxidize urea under the experimental conditions. Another reason could be that during the activation step, Ni^2+^ was formed as the dominant nickel redox state, so there were not enough Ni^3+^ sites to catalyze electrooxidation of urea. It should be noted that the sensor was also tested in a mixed solution containing organic compounds such as glucose. Data analysis indicated there was not a similar trend in reduction currents in the presence of glucose. The possible reason could be that glucose consumed Ni^3+^ under the applied potential for these experiments, converted it to Ni^2+^, and therefore interfered with the sensor response. However, with a slight change in the procedure, this issue could be solved, and a calibration curve based on the oxidation current could be developed, which is not discussed in this paper. Therefore, the nickel-based sensor with the above-mentioned procedure is suitable when there is no organic compound in the solution; otherwise, the procedure to develop calibration curves will be slightly different. It is noteworthy that the calibration curve in Fig. 8 was developed based on the given parameters for untreated blackwater, so for other environments the sensor could be recalibrated by having an estimate of the target parameters.

In order to validate the calibration curve developed for the simulated environment, mixed solutions were prepared based on the chemical composition introduced in Table 4 and various pH values in the pH range of 7–9. Then, the sensor was used to measure the current of these solutions following the procedure explained in Section 2.2. Based on the measured currents, the pH was calculated using the calibration equation shown in Fig. 8. As a reference method, the commercial glass electrode was also used to measure the pH of these solutions. Table 5 shows the experimental results of pH measurements made by these two methods for four solutions. As it can be seen, RSD% of the pH measurements by the nickel-based sensor is lower than 5%, confirming repeatability of the experiments.

**Table 5:** pH measurements using the glass electrode and nickel-based sensor in mixed solutions. Mixed solution constituents and composition are shown in Table 4.

Glass electrode	Nickle-based sensor	
pH	pH	Relative standard deviation %
7.33	7.19	3.90
7.97	7.24	4.42
8.57	8.08	3.60
8.8	8.46	4.08

The pH accuracy of the commercial pH meter is reported as ±0.01 pH, and the pH accuracy for measurements made by the nickel-based sensor was found to be an average of ±0.31. While, in terms of accuracy, the nickel-based sensor did not show an improvement over the traditional pH electrode; it could measure the pH very closely to the commercial pH meter. It can also overcome some of the glass pH electrode drawbacks. For example, it does not need an internal solution or a fragile glass membrane, so it could be applied in harsh conditions where a glass electrode is not applicable. It should be noted that we expect that optimizing the activation procedure and decreasing the test time could improve the pH accuracy of the sensor, which will be studied in future work.

### 3.4 The effect of cations on the sensor performance

The nickel-based sensor was employed to determine the pH of NaOH solutions to explore the role of the cations in the sensor current response. NaOH was used instead of KOH to prepare testing solutions and adjust their ${\mathrm{OH}}^{-}$ concentration and pH. The same procedure explained in Section 2.2 was followed to measure the current response. Then, the developed calibration curves for KOH solutions were used to find the pH of each NaOH solution. Table 6 demonstrates the values measured by the glass electrode and the nickel-based sensor. The nickel-based sensor can quantify the pH of NaOH solutions, suggesting different conductivity of ${\mathrm{Na}}^{+}$ and $K^{+}$ does not have a significant effect on the current and the calibration curves for one can be used for the other. The reason could be the strong catalytic activity of nickel and its reaction with hydroxyl ions.

**Table 6:** Quantification of pH of NaOH solutions using the calibration curves developed for KOH solutions.

Glass electrode	Nickle-based sensor	
pH	pH	Relative standard deviation %
7.90	7.99	0.49
8.92	8.42	0.33
9.83	9.48	0.22
10.90	11.46	0.2

### 3.5 Sensor stability

The long-term stability of the sensor was examined by applying the sensor for pH measurement of mixed solutions for a certain period. During this time, the sensor was stored in 0.01 M KOH in a closed beaker and under room temperature. Results show that the sensor under these conditions could be used daily for up to 9 months with the RSD < 7%. After this time, the working electrode should be polished or replaced.

## 4 Conclusion

In this study, we have successfully developed a nickel-based electrochemical sensor for direct ${OH}^{-}$ quantification and pH sensing. The sensing mechanism is based on the current produced from the reaction between the nickel electrode and the hydroxyl ions in solution. Analysis of chronoamperometric responses confirms a correlation between the current response and ${OH}^{-}$ concentration as well as the pH of the solution. The minimum detectable ${OH}^{-}$ concentration for the nickel-based sensor is 0.3 µM which is a significant accomplishment compared to previous methods. The other unique feature of this sensor is its wide detection range which is 0.3 µM to 4.8 M. Therefore, it is applicable in highly alkaline solutions where typical commercial glass electrodes do not operate adequately. The sensor has a rapid response time of 14 s and an RSD lower than 7% during 9 months of experiments. The measurements in mixed solutions proved the selectivity of the sensor towards ${OH}^{-}$. The sensor was also tested to determine the pH of common standard buffer solutions, and it demonstrated high repeatability and reliable results. The other advantage of this sensor is that conditioning and activation of the sensor are electrochemical and performed in the storage solution. As a result, the sensor will always be ready for measurements, and it is capable of remote sensing and online application. The electrochemical ${OH}^{-}$ sensor reported in this work offers several other attractive features. It does not need an internal solution and glass membrane as required by traditional glass electrodes, it does not need a separate reference electrode, and it provides a long lifetime. Moreover, its specific design could overcome miniaturization issues. With further modification of the procedure, the response time and repeatability of the method will be improved, and it could be validated for real applications such as water and wastewater quality monitoring. All the experiments for this study were performed at room temperature; future studies should include the effect of temperature as well as additional data analysis and optimization to increase sensitivity and accuracy.

## CRediT authorship contribution statement

**Behnaz Jafari:** Methodology, Formal analysis, Investigation, Writing - original draft. **Madhivanan Muthuvel:** Methodology. **Gerardine G. Botte:** Conceptualization, Formal analysis, Writing - review & editing, Project administration, Funding acquisition.

## Declaration of Competing Interest

The authors declare that they have no known competing financial interests or personal relationships that could have appeared to influence the work reported in this paper.
